# Clinical Efficacy of a Spray Containing Hyaluronic Acid and Dexpanthenol after Surgery in the Nasal Cavity (Septoplasty, Simple Ethmoid Sinus Surgery, and Turbinate Surgery)

**DOI:** 10.1155/2014/635490

**Published:** 2014-07-01

**Authors:** Ina Gouteva, Kija Shah-Hosseini, Peter Meiser

**Affiliations:** ^1^Institute of Medical Statistics, Informatics and Epidemiology, University Hospital of Cologne, Lindenburger Allee 42, 50931 Cologne, Germany; ^2^Ursapharm Arzneimittel GmbH, Industriestraße 35, 66129 Saarbrücken, Germany

## Abstract

*Background*. This prospective, controlled, parallel-group observational study investigated the efficacy of a spray containing hyaluronic acid and dexpanthenol to optimise regular treatment after nasal cavity surgery in 49 patients with chronic rhinosinusitis. *Methods*. The control group received standard therapy. Mucosal regeneration was determined using rhinoscopy sum score (RSS). Pre- and postoperative nasal patency was tested using anterior rhinomanometry. The participants were questioned about their symptoms. * Results*. Regarding all RSS parameters (dryness, dried nasal mucus, fibrin deposition, and obstruction), mucosal regeneration achieved good final results in both groups, tending to a better improvement through the spray application, without statistically significant differences during the whole assessment period, the mean values being 7.04, 5.00, 3.66, and 3.00 (intervention group) and 7.09, 5.14, 4.36, and 3.33 (control group). No statistically significant benefit was identified for nasal breathing, foreign body sensation, and average rhinomanometric volume flow, which improved by 12.31% (control group) and 11.24% (nasal spray group). * Conclusion*. The investigational product may have additional benefit on postoperative mucosal regeneration compared to standard cleaning procedures alone. However, no statistically significant advantage could be observed in this observational study. Double-blind, controlled studies with larger populations will be necessary to evaluate the efficacy of this treatment modality.

## 1. Introduction

Ventilation, mucociliary transport, and the epithelial barrier are considerably impaired initially following surgical procedures of the nasal cavity.

Injury of the nasal mucosa involved not only with the procedure but also prior to surgery caused by various pathologies usually results in the reduction of the protective secretion film and damage to the highly sensitive cilia [1]. Rapid wound healing from rhinosurgical procedures therefore reduces the risk of new infections considerably.

Although minimally invasive endoscopic technology and instruments enable functional endoscopic paranasal surgery that is gentle to the mucosa, the final results remain dependent upon proper wound healing of the nasal or paranasal mucosa without extreme scarring. Large-scale crusting, mucosal changes, ventilation disorders due to excessive secretion and edema, secondary hemorrhaging, or the development of synechia with possible reobstruction are critical factors that can lead to postoperative complications.

Besides frequent check-ups and wound debridement on the part of the treating physician, meticulous postoperative care of the mucosa using nasal irrigation, inhalation, sprays, and ointments on the part of the patient complements local treatment approaches and the measures taken to prevent adhesion up to the full and proper healing of the wound. In this connection, no established gold standard exists.

At present, investigations are available on a broad spectrum of topical nasal preparations for the postoperative care of the mucosa. To date, however, studies on the combination of hyaluronic acid (HA) and dexpanthenol as the main components of a nasal spray have not yet been carried out.

Hyaluronic acid belongs to the group of glycosaminoglycans and is an omnipresent macromolecule in the interstitium of vertebrates. It is involved in the modulation of various physiological processes (including morphogenesis, regeneration, wound healing, and tumor invasion [2]) and also controls signaling pathways, ergo cell behavior and interactions [2–4].

If tissue continuity is disrupted because of injury, a relatively uniform inflammatory response is induced in the body to break down necrotic tissue, eliminate pathogenic microorganisms, and restore initial integrity through tissue proliferation and repair [5, 6]. As a fundamental component of the extracellular space, hyaluronic acid functions as a framework for wound healing. In addition, it performs other various functions during the regeneration process. Its involvement and specific interaction in subprocesses are complex and to some extent still unknown for the individual steps to be attributed to a specific property.

As a response to tissue injury in the skin, the unusually high hyaluronic acid level influences tissue hydration during the subsequent inflammatory process. This is relevant with regard to cell proliferation and migration, as the pronounced hygroscopy of the polymer increases the moisture content of the tissue locally, which weakens cell adhesion mechanisms in the extracellular matrix and permits temporary separation for the purpose of cell migration and proliferation [7].

Scarless regeneration in human fetal wounds is attributed to unusual hyaluronic acid abundance in the matrix during embryonic development [8, 9].

Diverse biological effects of hyaluronic acid are related to its molecular size. High molecular sized polymers have antiangiogenic and immunosuppressive functions, thereby reflecting intact tissue, while smaller units are distress signals and potent inducers of inflammation, angiogenesis, and mobilization of immune cells [10–12]. Hyaluronic acid and its degradation products originating from the wound healing process are able to regulate tissue or cellular reactions, most notably the promotion of fibroblast proliferation and angiogenesis [2, 13].

The unique viscoelasticity and mucoadhesive capability of hyaluronic acid [14, 15] together with its high immunological and toxicological product safety have led to its versatile use in a number of application forms for various dermatological [16–22], pharmaceutical [3, 14, 23–32], and tissue engineering [33, 34] purposes, or during surgical procedures as well as for postoperative treatment [35–56].

In support of the therapeutic potential of sodium hyaluronate, hysan* Pflegespray* also contains dexpanthenol, which is a long-established active substance having excellent skin tolerance and penetration capacity [57] and a particularly positive impact on the mucociliary clearance of the respiratory epithelium [58, 59].

In the skin, dexpanthenol (provitamin B5) metabolizes to pantothenic acid (vitamin B5), which is essential for the normal function of the epithelial cells, especially during the energy-intensive early phase of epithelial regeneration (within the first 4 days) [60].

Particularly as a topical dermatological preparation for treating wound healing disorders, dermatoses, scars, extensive burn wounds, or skin transplantations [57, 60–62] as well as for treating wounds following nose surgery, the long-established anti-inflammatory and epithelium-protective effect [63] of dexpanthenol has been used for decades in clinical routine [63–66]. Various studies have scientifically confirmed the effectiveness of its preservative-free nasal ointment (predominantly) or spray application forms in treating rhinitis sicca anterior or after nasal and paranasal surgery [63–69]. It also improves the tolerability of rhinological preparations containing preservatives [1, 66, 69]. The local application of dexpanthenol in acute and chronic rhinitis is a part of routine standard therapy [66].

Corresponding to clinical experience, external therapy with dexpanthenol preparations is normally considered very well tolerated, having a minimum risk of skin irritations or sensitization [70].

Even though hyaluronic acid and dexpanthenol have long been clinically proven to be antiadhesive and mucosal conditioning substances separately, no study has yet investigated the possibility of a more intensive, wound-healing promotive effect based on the synergy of their set combination in a nasal spray. This was the reason that this dual-center, clinical trial examined a CE-labelled medical device (nasal spray) which was used for its intended purpose of regenerating damaged nasal mucosa; the study was carried out in strict accordance with the definition of nonintervention [71].

## 2. Patients and Methods

### 2.1. Patients

Included in the study were patients who suffered from chronic rhinosinusitis and who had undergone the following surgical procedures of the nasal cavity: septoplasty, simple ethmoid surgery, turbinate surgery, pansinus surgery, and maxillary sinus surgery.

The total population consisted of 49 patients. Of these, 27 patients were assigned to the intervention group. The other 22 patients comprised the control group which received customary conditioning preparations that were not documented.

### 2.2. Design

This trial was carried out as a prospective, open-label, observational study in two doctor's offices from 11 September 2008 to 13 September 2011. Investigators collected test results and the patients' subjective assessments at a minimum of five check-up visits, the initial examination, three intermediate examinations, and one final examination, and documented the data in the observation form.

At the initial examination, the patient was thoroughly informed about the planned noninterventional study, indications for surgery, and preoperative rhinomanometry. Patients were not randomised to receive the study medication. The choice of the appropriate postsurgical care was based on the investigator's judgement of the patients' clinical condition after surgery and the patients' willingness to apply the spray regularly instead of using the alternative nasal pipetting or ointments.

All participating patients signed a data privacy declaration form, giving their consent to allow their data in pseudonymous form to be recorded and forwarded to the sponsor or competent authorities.

This observational study examines how wound healing is influenced after the first check-up and after removal of packing material, if inserted. This was not documented in the observation form and was not considered in the results.

Furthermore, the adjuvant postoperative administration of antibiotics, antiphlogistics, or analgesics as concomitant medication, if necessary, was recorded.

A repeated anterior rhinomanometry (at visit 1 in the 1st postoperative week) and anterior rhinoscopy (optional endoscopy) (at all other visits in accordance with the observation schedule) were conducted for documentation purposes, for monitoring the final results of surgical treatment with respect to nasal patency, and for the visual assessment of nasal mucosa conditions. In addition, the patients were questioned about their subjective perceptions with respect to nasal breathing and foreign body sensation, tolerability of the nasal spray, and any noticeable problems or complaints in connection with the preparation used.

This paper was compiled in accordance with the STROBE (strengthening the reporting of observational studies in epidemiology) statement.

### 2.3. Ethical Aspects and Professional Regulations

The investigational preparation and the control medication were both CE-certified. According to the Medical Devices Act, this investigation was therefore exempted from requiring approval from the competent federal authority and the competent ethics committee. Investigators in charge of the study received consultation with respect to professional regulations before the study commenced.

### 2.4. Study Medication

The object of the investigation was the nonprescription “hysan Pflegespray” manufactured by Ursapharm Arzneimittel GmbH, Saarbrücken, Germany. At the beginning of the study (2008), the product was called “Hylocare-Nasenspray.” It was renamed “hysan Pflegespray” in 2011. It is a liquid pharmaceutical preparation with a dosing spray applicator for the prophylactic or curative topical treatment of inflammatory conditions. It can be applied as monotherapy as well as a concomitant adjuvant to therapy with decongestant nasal sprays (or drops) for rhinosinusitis.

Hysan Pflegespray is a sterile, preservative-free solution containing 0.25 mg/mL sodium hyaluronate, 2% dexpanthenol, as well as sodium dihydrogen phosphate × 2H_2_O, sodium monohydrogen phosphate × 2H_2_O, sorbitol, and water. One bottle contains 10 mL of solution, which corresponds to approximately 70 sprays [72].

### 2.5. Dosage of Study Medication

One to two puffs of nasal spray to each nostril were to be administered three times, distributed evenly throughout the day. If additional therapy with other nasal sprays was applied, nasal spray was always to be used last, allowing at least 30 minutes to elapse between nasal sprays.

### 2.6. Conventional Care Preparations

Treating otolaryngologist Nr. 1 administered a proprietary composed solution for pipetting by the patients 3-4 throughout the day in both nasal cavities, with the following ingredients: glucose-monohydrate: 5.0 g, menthol: 0,025 g, Olynth 0.1% nasal drops (active agent: xylometazoline hydrochloride): 5.0 g, eucerinum anhydricum: 7.0 g, and peanut oil: ad. 50.0 g.

Treating otolaryngologist Nr. 2 prescribed a proprietary composed ointment as a standard local postoperative care formulation which was to be applied twice a day. The ingredients of the mixture were hydrocortisone: 0.01 g, vitamin A (retinoic acid): 0.4 g, Bepanthen ointment (active agent 5% Dexpanthenol): 16.0 g, and Otrivin nasal drops 0.1% (active agent: xylometazoline hydrochloride): 1 g.

Both preparations were individually compounded by a pharmacist.

### 2.7. Recording of Efficacy

The primary variable was changed in the sum score (RSS: rhinoscopy sum score), which was attained from the clinical, objectively recorded endoscopy findings: nasal dryness, dried nasal mucus, fibrin deposition, and nasal obstruction. All variables pertaining to rhinoscopic mucosal findings were evaluated using a 4-point scale as follows: absent = 0, mild = 1, moderate = 2, and severe = 3.

For the secondary variable, the patient's subjective perception of unobstructed nasal breathing and foreign body sensation was rated on a scale from 1 to 3 (1 = good, 2 = average, and 3 = poor).

In order to categorize the initial symptom situation and objectivization of patients' statements on symptoms of nasal obstruction and to record the effectiveness of surgery and the monitoring of the final results of surgery, pre- and postoperative active anterior rhinomanometry were carried out at the initial examination.

The treating physician recorded general efficacy and tolerability in free-text format at the end of the observation period.

### 2.8. Recording of Safety

In spite of the broad clinical experience with both active ingredients contained in nasal spray, special importance was attached to the documentation of adverse events when collecting data in the present study.

The holder of the marketing authorization for the medical device investigated here was obliged to report the severity or intensity of adverse events to the Bundesinstitut für Arzneimittel und Medizinprodukte (The Federal Institute for Drugs and Medical Devices, Pharmacovigilance Division). The treating physician was also able to document his/her own or the others comments or noticeable signs relating to the patient's general condition, product use, or general remarks about the course of treatment in free-text format on the observation forms under “Other notes.”

## 3. Analysis

### 3.1. Handling of Documentation Errors and Analysis Problems

Missing entries resulted in incomplete data sets, so individual parameters could not be evaluated. These missing data were generally treated as “missing values” and not taken into consideration in the analysis.

### 3.2. Statistics

The detailed analysis of the parameters was carried out using SPSS 19 statistics software manufactured by SPSS Inc. Frequencies, mean values, standard deviations, medians, and minimum and maximum values within the treatment groups were given for the various variable forms.

To this purpose, patient data were first entered into separate SPSS databases by two people independent of each other. The monitoring person in charge recognized all occurring discrepancies and illogical values. The merged database underwent a plausibility test. Data were then synchronized and subsequently analyzed in SPSS. A significance level of *α* = 0.05 was defined for all statistical tests.

### 3.3. Data Analysis

Descriptive statistical methods were applied. The statistical values (number, mean value, minimum, maximum, and standard deviation) for continuous variables such as height, age, and time periods were listed in a table. Discrete variables were categorized in the form of frequency distributions with their percentage-wise relationships to the total sample. Free-text answers were transferred post hoc in the appropriate coding schemes and analyzed as frequency distributions. Clinical parameters of disease progression were evaluated and illustrated in the form of intraindividual differential analyses (first versus last examination). Categorically recorded clinical data were analyzed in the form of contingency analyses (before/after). Subgroup analyses were not defined a priori. Any results yielded using comparative statistical methods were of purely explorative character.

## 4. Results

### 4.1. Patients: Demographic Data

Overall, 49 patients participated in the study, 8 of whom were female and 41 male. Patients were aged 15 to 58 years (mean age of the total population was 33.12 years, SD: ±11.04 years).

### 4.2. Rhino-/Endoscopic Mucosal Findings

**Figure 1:**
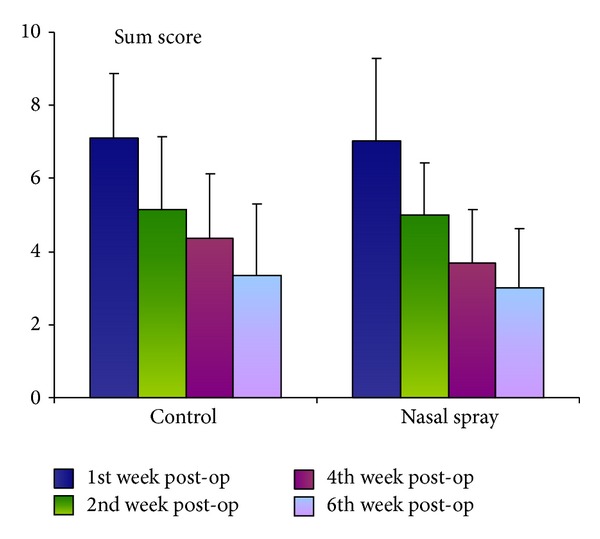
Rhinoscopy sum score (RSS) of the individual groups.

A scale from 0 to 3 (0 = none, 1 = mild, 2 = moderate, and 3 = severe) was used to assess all parameters (nasal dryness, dried nasal mucus, fibrin deposition, and development of obstructions). The treating physician thereby documented, added, and averaged the mucosal findings obtained via rhinoscopy/endoscopy during the weeklong application of the nasal spray. The resulting rhinoscopy sum score (RSS) is shown in Table 1 and illustrated in Figure 1. Details for each individual parameter can be found in Table 2.

### 4.3. Patient Evaluation of Nasal Breathing and Foreign Body Sensation

A scale from 1 to 3 (1 = good, 2 = moderate, and 3 = poor) was used for the patients' self-assessment of nasal breathing. The patients rated their subjective perception of a foreign body during the entire postoperative follow-up interval on a scale from 0 to 2 (0 = absent, 1 = moderate, and 2 = severe).

### 4.4. Pre- and Postoperative Rhinomanometry (8th–10th Postoperative Day)

**Figure 2:**
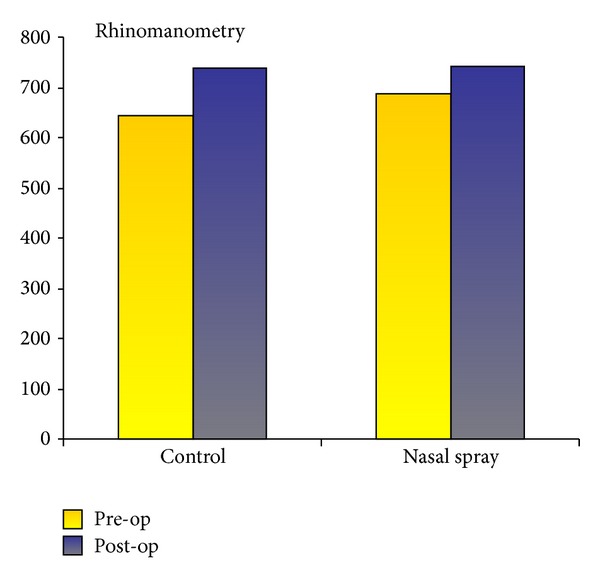
Values for pre- and postoperative rhinomanometry in [mL/s] at 150 Pa, sum left and right nasal cavity. Bar graph.

The comparison between pre- and postoperative rhinomanometry shows a similar percentage increase for the mean volume flow in both comparison groups: hysan group 11.24% and control 12.31% (Table 4, Figure 2). Here, the mean preoperative volume flow of 688.13 mL/s (±209.524 mL/s) in the hysan group was slightly above the initial value for the control group at 643.16 mL/s (±188.253 mL/s).

## 5. Discussion

In spite of gender inhomogeneity, both patient populations were comparable.

It was up to the treating physician to decide whether the patient required concomitant medication in the postoperative healing period. No valid conclusion could be made with regard to the possible influence of antibiotics, antiphlogistics, or analgesics on the effect of hysan nasal spray.

In both patient populations, the condition of the endonasal mucosa improved continuously during the follow-up period with respect to the defined objective parameters (nasal dryness, dried nasal mucus, fibrin deposition, and obstruction). The mean RSS values, however, exhibited no significant differences between both populations (see Table 1). Nevertheless, the hysan group showed lower values in the 4th and 6th week (3.66 pts. and 3.00 pts., resp.) compared to the control group (4.36 pts. and 3.33 pts., resp.). A clinical comparison of an isotonic saline spray containing dexpanthenol with a simple saline spray for postoperative treatment over 6 weeks showed comparable efficacy regarding all objective parameters of the endoscopic mucosal analysis and the majority of subjective symptoms as well [59]. Another similarly designed study compared an ointment containing hyaluronic acid (Rhinogen) with a plant-based ointment (H.E.C.). Both preparations, yet again, did not differ significantly with respect to the objective parameters of mucosal dehydration, formation of blood clots, and mucosal lesions [73].

Furthermore, the application of the control substances might have led to falsified results in the control group, because of the potentially positive effects of their active agents on the wound healing process. An enhancing effect on morphological and functional cilia regeneration has been ascribed to retinoic acid [74–77]. Systemic prednisolone administration together with local application of 5% of dexpanthenol ointment had a beneficial effect especially on the late spontaneous wound closure in a standardized animal model [68]. On the other hand, the addition of dexpanthenol (5%) resulted in a statistically significant reduction of the toxicity of *α*-sympathomimetic decongestants like xylomethazoline [63].

Mean values for the rhinoscopic parameter of nasal dryness were almost identical in both groups of this observational study except in the 4th postoperative week (Table 2). In the control group, the mean value at visit 2 (1.24 pt.) dropped only slightly by visit 3 (1.21 pt.), while it decreased more in the intervention group (from 1.25 to 1.00 pt.: no statistical significance). This fact could be attributed to the intense hydration effect of hyaluronic acid. This explanation was based on the important clinical observations made by Soldati et al. that the application of the hyaluronic acid containing ointment prevented large-scale crusting in the first postoperative week compared to the control substance [73].

The mean values for the parameter dried nasal mucus exhibited almost analogous dynamics (Table 2). Although a greater decrease in the mean value for the degree of dried nasal mucus was observed among hysan users in the 4th and 6th weeks, it did not reach a level of significance. This was probably ascribed to the increased local hydration due to hyaluronic acid, which formed an even, stable, and long-lasting moisture film on the nasal mucosa, thereby serving as a lubricant during the vulnerable regeneration process and as a vehicle for dexpanthenol in the late phase of wound healing phase, allowing its full cilia-protective effect to unfold. Consequently, the improved mucociliary clearance helped gently loosen dried nasal mucus.

The significantly decreased crusting during the 1st and 2nd postoperative weeks after applying a combination solution for mucosal care (that contained isotonic saline, algae extract, hyaluronic acid, panthenol, and Tonimer Gel Spray) may also support this assumption [78]. Another clinical comparison between dexpanthenol seawater spray (Mar plus) and normal saline irrigation resulted in less crusting at the 2nd check-up visit and better mucociliary clearance at the 4th check-up in the intervention group [79].

A clinical reduction in the formation of dried nasal mucus was observed after an 8-week treatment with dexpanthenol in a spray application form in patients with chronic rhinitis sicca as well [65]. Analogous results were shown by Hahn et al. after the four-week application of a dexpanthenol ointment [80].

The mean values for fibrin deposition in the hysan group were somewhat lower in the late phase of wound healing between the 4th and 6th postoperative weeks compared to the prior weeks (Table 2).

The last mucosal findings collected by the treating physician concerned nasal obstruction as observed via rhinoscopy. At all scheduled examinations, the results of both groups were of similar magnitude and without statistically significant differences (Table 2). Notable were the initially rapid drop of the mean value in the hysan group in the 2nd week and the consistent small decline over the remaining three visits. This tendency gave rise to the presumption that the use of the nasal spray greatly reduced nasal mucosal obstruction in the early phase of wound healing and was responsible for lower mean values in general over the entire period of application. In the end, however, no considerably better results were obtained than in the control group.

This correlation could lead one to assume that, due to hyaluronic acid, increased tissue hydration, which according to Kühnel et al. enables the early reduction of dried nasal mucus [81], and the reduced formation of hyperplastic granulation tissue [68, 82], as well as the accelerated reepithelialization due to dexpanthenol [82], resulted synergistically in diminished postoperative nasal obstruction symptoms.

Our study, however, could not clearly verify this theory.

The subjects were asked to categorize their subjective perceptions of free nasal breathing and foreign body sensation on the observation form, since according to definition an observational study is to consider the individual assessments of the product users as an important influencing factor. The results are summarized in Table 3.

The responses from the nasal spray patients at the first two examinations are striking with their low mean values for free nasal breathing, again without significant differences to the control group. The results were almost identical for the last visits in both groups (Table 3). This fact suggests that patients tended to perceive nasal breathing as freer while using hysan spray during early postoperative tissue regeneration. A possible explanation for this could be the film formed by the aerosol of the nasal spray that temporarily covered the mucosal areas and which the patient mistakenly interpreted as mildly impaired nasal breathing in the last two weeks.

Similar results of positive influence of an isotonic seawater spray containing dexpanthenol on the total nasal subjective symptom score and on patient satisfaction, although again without significance, were confirmed by Fooanant et al. [79].

In this connection, conflicting results have been mostly published in the literature. A significant improvement of the patient-reported comfort (ease of breathing, nasal tension, and feeling of dryness) has been observed by Ercan et al. [78]. Soldati et al. also confirmed a significant improvement in respiration among subjects who applied ointment containing hyaluronic acid, with nasal patency being highly significant on the 7th postoperative day and significant on the 14th postoperative day [73]. Kehrl and Sonnemann [65] and Hahn [80] verified positive dynamics in the subjective sensory scale in terms of nasal airway obstruction among dexpanthenol spray users with rhinitis sicca. Significant improvement of nasal obstruction showed a saline aerosol containing hyaluronic acid in the phase of functional regeneration during sinonasal remodeling, as described by Macchi et al. [83].

The mean value for patient's self-assessment of foreign body sensation was in general relatively positive (Table 3). Limitations existed with respect to the time at which this symptom appeared (the parameter was not enquired upon at every scheduled examination but instead globally assessed for the entire postoperative interval). Contrary to expectations, at a value of 0.52 points in the control group it tended to fall into the category “not present,” and at 0.71 points, the parameter tended slightly to “moderately pronounced” among hysan users. One reason for this could be the protective film on the mucosa as mentioned earlier that compromised the patients' perception of a foreign body.

Rhinomanometry data confirmed volume enlargement of the nasal cavity by 12.31% in the control group and by 11.24% in the intervention group, the difference not being statistically significant (Table 4). The application of the test substance (period: 8th–10th postoperative day) resulted in no significant, objectively measured reduction in nasal resistance. Volume enlargement of the nasal cavity relies in fact only on structure-reducing measures which successfully eliminated any obstruction to nasal breathing.

The investigator's impressions concerning the good tolerability and efficacy of the preparation agreed to a large extent with those of the patients and with the literature.

Soldati et al. also reported high acceptance, safety, and tolerability of the ointment containing hyaluronic acid. Worth mentioning, besides the positive organoleptic evaluation relating to the smell and the sensation of cooling upon application, was also the absence of adverse reactions [73]. The study conducted by Fooanant et al. yielded similarly good results for the dexpanthenol spray with respect to effectiveness and patient satisfaction [79]. The positive influence of dexpanthenol preparations on the subjective symptoms in patients with rhinitis sicca [65, 80] and their high acceptance were, yet again, able to confirm the clinically relevant and statistically significant superiority of the substance.

Dropouts were the most common subject appearing in the text field “other doctor's comments.” Only one mild irritation was noted, probably due to intolerance of one of the ingredients; no entry, however, was made under the item “adverse events” on the observation form.

## 6. Conclusion

Surgical procedures of the paranasal sinuses leave behind extensive wounds that are left up to the secondary self-healing process [6, 84]. The aim of postoperative treatment is optimum wound healing with minimal morbidity.

The present limited observational study showed that the nasal spray is a safe preparation for care of the mucosa after rhinosurgical procedures. Its use did not negatively affect postoperative mucosal regeneration, yet no significant improvement of mucosal conditions could be observed either.

The results might have been impaired by the two treating physicians, who might have not always assessed the nasal mucosa conditions identically, by the possible positive influence of the active ingredients of the conventional care preparations on the control group, or by the unbalanced concomitant use of antibiotics and anti-inflammatory medication. Furthermore, the conclusions of the study might have somewhat been affected by the limited participants number, by the lack of randomization and blinding, by the heterogeneity of the initial pathology state among the patients, or by the variety of the surgical procedures that are scarcely comparable.

Additional multicenter, double-blind studies with larger populations, having a comparable degree of pathology and extent of mucosal extirpation in the same surgical procedure along with detailed surveys, are necessary to clarify further aspects of postoperative wound healing processes of the respiratory epithelium and the influence thereof for achieving adequate functional regeneration and better quality of life.

## Figures and Tables

**Table 1:** Rhinoscopy sum score (RSS), postoperative. Confidence interval 95%.

		RSS week 1	RSS week 2	RSS week 4	RSS week 6
Control group	*N* (valid)	22	21	19	18
	Mean	**7.09**	**5.14**	**4.36**	**3.33**
	Standard deviation	1.77037	1.98206	1.77045	1.97037

Nasal spray group	*N* (valid)	24	24	21	19
	Mean	**7.04**	**5.00**	**3.66**	**3.00**
	Standard deviation	2.23566	1.41421	1.49443	1.63299

**Table 2:** Individual rhinoscopy findings during the examination period (1st–6th postoperative week).

Post-op week		“Dryness”				“Dried nasal mucus”				“Fibrin deposition”				“Obstruction”			
		1.	2.	4.	6.	1.	2.	4.	6.	1.	2.	4.	6.	1.	2.	4.	6.
Control group	*N* (valid)	22	21	19	18	22	21	19	18	22	21	19	18	22	21	19	18
	MV	**1.64**	**1.24**	**1.21**	** 0.83**	**1.95**	**1.33**	**1.16**	**0.89**	**1.86**	**1.33**	**1.11**	**0.89**	**1.64**	**1.24**	**0.89**	**0.72**
	SD	0.581	0.539	0.535	0.514	0.653	0.658	0.501	0.583	0.468	0.483	0.567	0.583	0.581	0.539	0.459	0.575

Nasal spray group	*N* (valid)	24	24	21	19	24	24	21	19	24	24	21	19	24	24	21	19
	MV	**1.67**	**1.25**	**1.00**	**0.84**	**1.92**	**1.29**	**0.95**	**0.74**	**1.83**	**1.42**	**0.90**	**0.74**	**1.63**	**1.04**	**0.81**	**0.68**
	SD	0.702	0.442	0.316	0.375	0.717	0.464	0.384	0.452	0.702	0.504	0.436	0.452	0.576	0.359	0.602	0.478

**Table 3:** Patient evaluation of nasal breathing and foreign body sensation.

Postoperative week		Nasal breathing				Foreign body sensation
		1.	2.	4.	6.	1–6.
Control group	*N* (valid)	21	21	19	18	21
	Mean value	**1.90**	**1.48**	**1.11**	**1.00**	**0.52**
	SD	0.625	0.512	0.315	0.000	0.512

Nasal spray group	*N* (valid)	24	24	20	19	24
	Mean value	**1.58**	**1.25**	**1.10**	**0.95**	**0.71**
	SD	0.584	0.442	0.447	0.229	0.464

**Table 4:** Values for pre- and postoperative rhinomanometry in [mL/s] at 150 Pa, sum left and right nasal cavity.

		Preoperative rhinomanometry sum left and right at 150 Pa in [mL/s]	Postoperative rhinomanometry sum left and right at 150 Pa in [mL/s]	Delta rhinomanometry	Improvement in %
Control group	*N* (valid)	19	18	16	
	Mean value	**643.16**	**737.67**	**79.19**	**12.31**
	SD	188.253	118.457	179.300	

Nasal spray group	*N* (valid)	23	19	18	
	Mean value	**688.13**	**743.05**	**77.33**	**11.24**
	SD	209.524	140.956	206.292	
